# Effects of Spectral Ranges on Growth and Yield in Vertical Hydroponic–Aeroponic Hybrid Grow Systems for Radishes and Turnips

**DOI:** 10.3390/foods14111872

**Published:** 2025-05-24

**Authors:** Adia Shadd, Nima Asgari, Joshua M. Pearce

**Affiliations:** 1 John M. Thompson Centre for Engineering Leadership and Innovation, Western University, London, ON N6A 5B9, Canada; 2 Department of Electrical & Computer Engineering, Western University, London, ON N6A 5B9, Canada; 3 Ivey Business School, Western University, London, ON N6G 0N1, Canada

**Keywords:** controlled environment agriculture, CEA, vertical farming, LED, PPFD, root vegetable, turnip, radish, agrivoltaics

## Abstract

As climate change destabilizes food crop production, there is a growing interest in controlled environment agriculture (CEA). Although light-emitting diodes (LED) have made CEA economically viable for some high-value crops when coupled to agrivoltaics (solar photovoltaics + agriculture), it has generally not been used for root vegetables. This is the first study to demonstrate that radishes and turnips could be grown in a reasonable period of eight weeks in an agrivoltaic agrotunnel using both lighting and grow walls optimized for lettuce growth. As reduction in LED energy use is important to minimize capital costs for solar energy, this study investigated three lighting treatments (red, white, and full-spectrum as control). The normalized yields (adjusted for total energy provided by each treatment) showed that both cultivars preferred red light, and harvested green leaves provided higher masses than the roots, although turnips appeared to be far more adaptable to vertical growth than radishes (>450% for roots and >50% for leaves per pot compared to radishes for the control treatment). The results show promise for providing true net-zero carbon emission root vegetables year-round with similar agrivoltaics-powered CEAs. Future work is needed with light intensity trials to optimize light recipes.

## 1. Introduction

Indoor farming or controlled environment agriculture (CEA) has been a rapidly growing research topic due to both climate destabilization [1] leading to increased conventional agriculture inconsistency as well as new technological developments like high-efficiency light-emitting diode (LED) lighting [2]. LEDs consistently outperform conventional artificial lighting for growth, yield, and nutritional content of various plants due to their optimized spectrum provision per energy consumed [3,4]. As LEDs allow for granular spectral tuning, research often centers around the spectral impacts on the growth, yield, and various nutritional contents of the resulting plants [5]. This work has resulted in a widely accepted result that red (625–700 nm) and blue (425–475 nm) light is required for ideal fresh mass production, and that supplemental green (475–625) and infrared (IR) (700–750 nm) lights also improve the photosynthesis and health level of some crops [6]. In this regard, several studies agree that optimal growth occurs with a blue–red ratio of 0.5 or higher [7,8,9,10]. It has been widely shown that far red light promotes total biomass and elongation [11], red light promotes biomass and reduces nitrate concentration [12], green promotes growth [13], and blue increases chlorophyll [12] and flowering [14]. In conclusion, plants grown under multiwavelength irradiation with optimized ratios between them will have higher photosynthetic activity, higher yields, and healthy growth [15,16]. The vast majority of these studies have focused on conventional horizontal growing systems and leafy vegetables. Recently, however, increases in yield per unit area have been observed with true vertical farming (vertically placed plants rather than horizontally layered hydroponic systems) in walls [17]. Systems similar to the agrivoltaics agrotunnel enable extremely high land utilization in vertical grow walls [18]. The agrivoltaics agrotunnel operates with conventional agrivoltaics (partially transparent solar photovoltaic (PV) systems providing shading for conventional outdoor agriculture), providing the electricity required to power heat pumps, water pumps, and LED lighting for a CEA tunnel [18]. The optimization of LEDs is particularly important for this type of CEA, as the capital cost of the system is dependent on the energy use, as the PV provides all the electric power for the CEA. For example, if part of the spectra (and thus energy) that is used for grow lights can be reduced, the overall size of the PV system necessary for net-zero production would also be reduced. The impact of spectral effects on these systems on the agrotunnel and the broader true vertical growing is relatively unexplored. Thus, the impacts of spectral lighting on CEA in vertical farming systems have not yet been verified.

To fill this knowledge gap, this study investigates the impact of three spectral light treatments on plant growth in vertical farming of relatively unexplored root vegetables: radishes and turnips [19]. Radishes have been minimally investigated, and turnips have not been investigated in the literature for the spectral effects of growing in a vertical farming system [20]. The literature often focuses on leafy vegetables due to their high concentration of calories, vitamins, minerals, fibers, and antioxidants, and for this reason, vertical farming has been largely developed with leafy vegetables in mind [20]. This priority introduces a challenge when studying root vegetables in these conditions, as features such as watering cycles and pot size are not intended for root vegetables and must be optimized manually. It is hypothesized that shifting the spectrum will impact growth, and this relationship may enable the identification of an optimal balance between energy consumption and growth, as previous studies on other crops have demonstrated similar effects. For this study, the baseline of information determined for the specific biological events activated by each wavelength range are explored for: designated red (620–700 nm), white (425–650 nm), and full spectrum or control (425–750 nm) light, as well as species’ varying responses to identical treatment. The results will be compared against prior studies and discussed in the context of using specific light recipes for individual crops to provide sustainable CEA.

## 2. Materials and Methods

### 2.1. Seeds

Seeds of Raphanus sativus (French Breakfast radish) (18280A Pkg, Veseys Seeds, York, PE, Canada) and Fuku Komachi (turnip) (19610A Pkg, Veseys Seeds, York, PE, Canada) were planted in rows of ports in an agrivoltaic agrotunnel (Food Security Structures Canada (FSSC), LO, Canada) [18]. Seeds that did not germinate were removed and discounted from this study. The total number of samples of each specimen are summarized in Table 1.

### 2.2. Agrotunnel Conditions

The agrotunnel was kept at a temperature of between 22 and 23 °C with a relative humidity of between 55% and 60%. The CO_2_ level in the grow room ranged between 600 and 1000 ppm during the cultivation period of the plants. For the radish and turnip, the target electrical conductivity (EC) was 1.8–2.4, and the target pH was kept at 6–6.5. The grow walls in the agrotunnel operate as a hybrid aeroponic–hydroponic system. This system is neither pure hydroponic (roots are submerged in nutrient water) nor pure aeroponic (roots are being sprayed with nutrient-rich water). The perforated peat pots had a porous structure and 70–30 mixture of coco coir and perlite as the main grow substrate inside, which could absorb and hold the nutrient water, which was pumped to the top of the walls and allowed to drip and cascade through the pots. The irrigation cycle occurred twice in 2 min watering durations each day. The 10-12-22 (N-P-K) ForaPro and 14-0-0 Calcium+Micros FloraPro nutrients were mixed with a ratio of 1:0.75 and used mainly to feed the crops’ roots. It is important to highlight that the cultivation period of both crops (from seed to harvest) was 8 weeks.

### 2.3. Grow Walls

**Figure 1:**
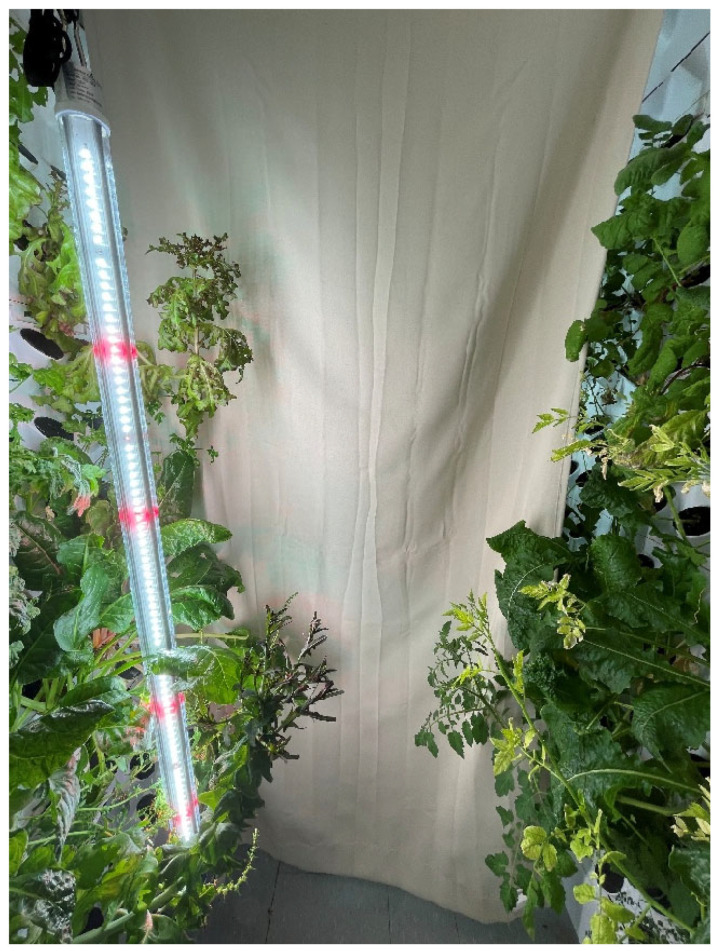
Curtain setup between walls with full-spectrum light.

**Figure 2:**
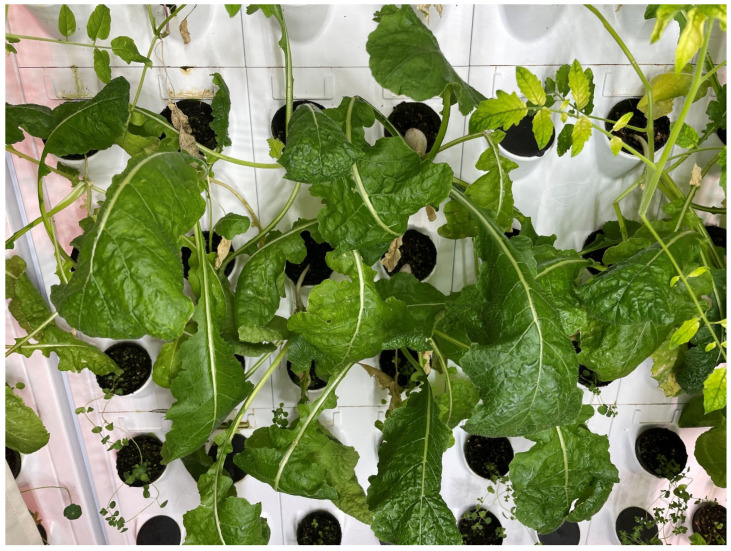
White light treatment wall with curtains on either side.

Two walls facing one another were planted with 10 rows of 24 ports, which were vertically divided into sections by curtains and covered such that only the applied wavelength of light and lights were adjusted to provide specific wavelengths to each section, as shown in Figure 1 and Figure 2.

### 2.4. Lighting

Lighting was provided by BGL 360A lights (FSSC, London, ON, Canada) and was operated for 24 h per day. The wavelengths in each section were measured with an Oceanview Ocean FX mini spectrometer and analyzed with Oceanview software v2.0.16 (Orlando, FL, USA) to ensure isolation of spectral conditions across different treatments. Based on the absolute irradiance provided by the Oceanview measurements, Equation (1) was used to find the energy of a single photon at a given wavelength (taken as the peak wavelength of each treatment):

(1) $$E_{photon}=\frac{hc}{\lambda }$$

where E is the energy of a single photon in Joules, h is Planck’s Constant (6.626 × 10^−34^ Js), and c is the speed of light (3.00 × 10^8^ m/s). The photon flux can be calculated using Equation (2):

(2) $$Photon\;Flux\left[photon/m^{2}/s\right]=\frac{Irradiance\left[J/m^{2}/s\right]}{E_{photon}\left[J/photon\right]}$$

Finally, the flux can be converted to micromoles using Equation (3).

(3) $$Photon\;Flux\;Density\left[\mu mol/m^{2}/s\right]=\frac{photon\;flux\left[photons/m^{2}/s\right]}{6.022\times 10^{17}\left[photons/mol\right]}$$

**Figure 3:**
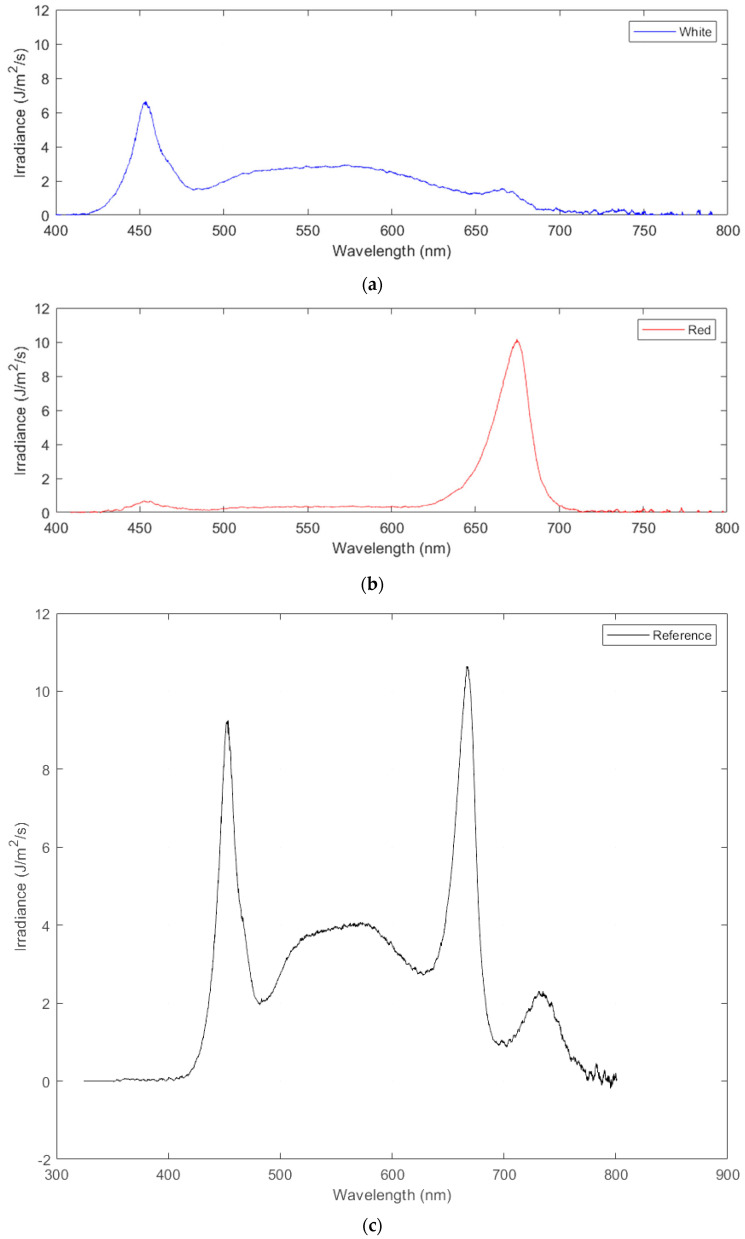
Irradiance as a function of wavelength for the spectral composition of (**a**) white light, (**b**) red light, and (**c**) full spectrum or control treatment.

Applying these formulas yields the following photosynthetic photon flux density (PPFD) values for each of the three treatments applied: red light only (620–700 nm) at a PPFD of 57 μmol/m^2^/s (Figure 3a), white light only (425–650 nm) with a PPFD of 63 μmol/m^2^/s (Figure 3b), and control light (full spectrum), for a combined PPFD of the control of 123 μmol/m^2^/s (Figure 3c).

Normalization of energy between treatments was used to better interpret the results, which was accomplished by similarly calculating the energy contribution from each treatment with Equation (1), integrated over the range of wavelengths characteristic to each treatment. These values were then compared to the total energy, and their percentage contribution was used to accordingly scale the height, leaf count, and yield of each crop by an appropriate ratio ($X_{norm}$ in Equation (4)). It was found that for the particular grow lights used, red contributed 28.9% of the control light, white was 70.2%, and the rest was made up of IR light.

(4) $$X_{norm}=X_{non-norm}\times \frac{100}{E_{\lambda ,share}}$$

Here, $E_{\lambda ,share}$ is the percentage contribution of each spectrum into the full spectrum of the grow lights, and X_non-norm_ is the agronomic value before normalization.

### 2.5. Measurements

Measurements taken included plants’ green leaf height (mm), leaf count, chlorophyll content (atLEAF Chloropyll meter, Wilmington, DE, USA), and fresh yield measured with a Starfrit digital scale with an uncertainty of ±0.1 g (Longueuil, QC, Canada). Measurements were taken for all plants in each treatment that were accepted to be within one standard deviation of the average (only one outlier for turnip data, which was affected by improper irrigation). The plants were grown and harvested over an 8-week period.

## 3. Results

The height and leaf counts are presented both as the raw results and as the normalized results (adjusted for total energy provided by each treatment). This was done to better approximate the growth that would be seen under equal light intensity conditions, which were not explicitly tested in this study.

### 3.1. Plant Heights

**Figure 4:**
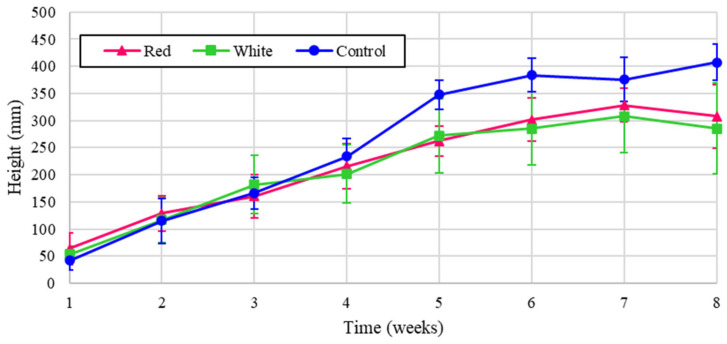
Turnip heights over eight weeks of growth.

**Figure 5:**
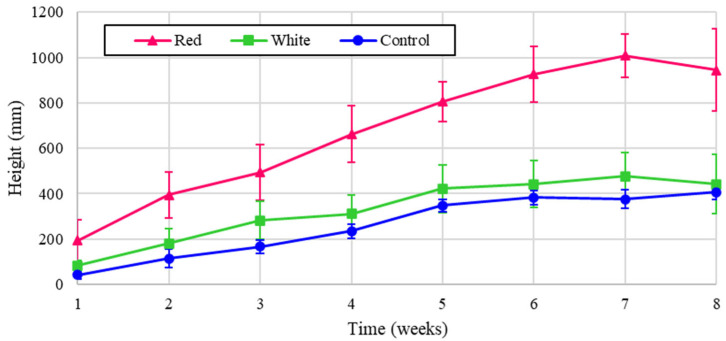
Turnip heights over eight weeks of growth (normalized energy from LEDs).

The experimental plant height is shown for turnips for the three light treatments in Figure 4 over the eight-week cultivation period, and the results normalized to energy heights are shown in Figure 5. Figure 4 shows that turnip height was the largest under the control, but when normalized for energy, the turnip height was substantially greater with red light. The proximity of the graphs for two treatments of red and white in Figure 4 reflects the critical influence of the red spectrum on plant height despite the lower energy contribution of this wavelength. This significance is shown in the dominant graph for the normalized analysis, shown in Figure 5.

**Figure 6:**
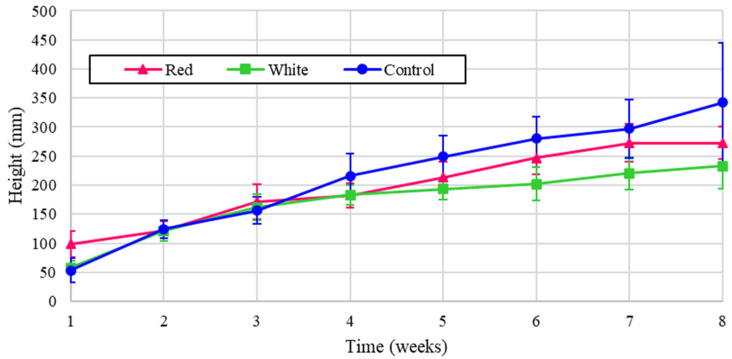
Radish heights over eight weeks of growth.

**Figure 7:**
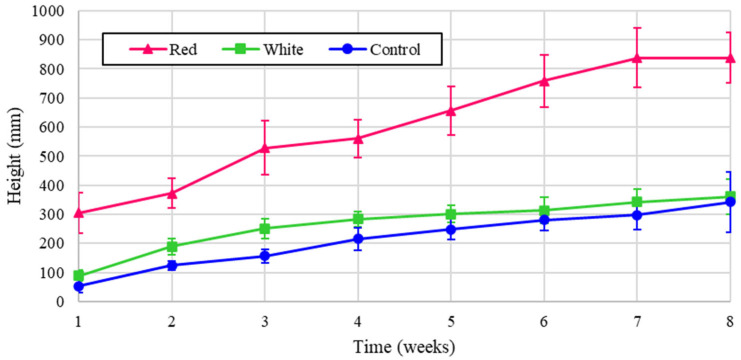
Radish heights over eight weeks of growth (normalized energy from LEDs).

Similarly, the height of the radish plants is shown in Figure 6, and the normalized values are presented in Figure 7. Although it may be expected that the control would outperform the other treatments as it did in Figure 6, radishes are also known to thrive under red light [7], and these results were confirmed by the normalized values shown in Figure 7.

These results indicate the that turnips had a similar response to the red light as the radishes.

### 3.2. Leaf Count

**Figure 8:**
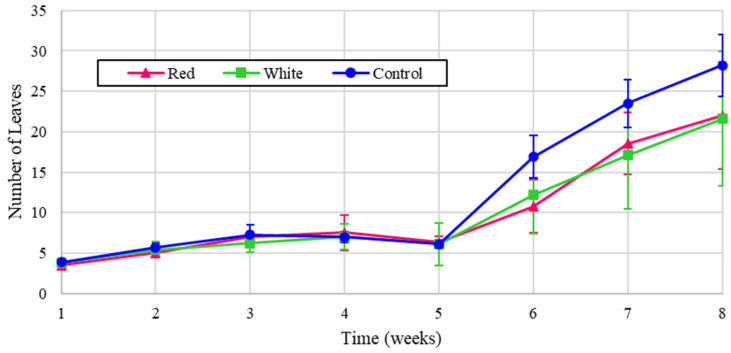
Number of turnip leaves over eight weeks of growth.

**Figure 9:**
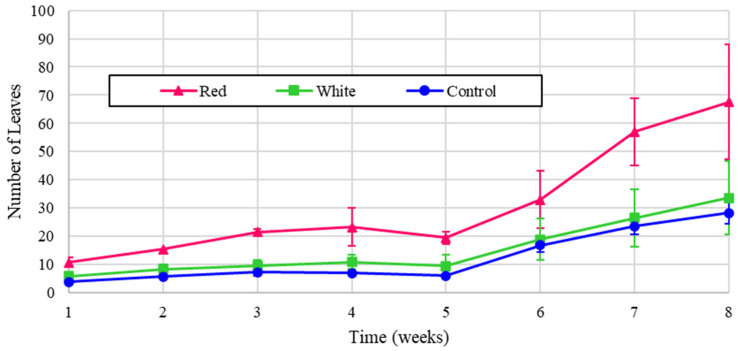
Number of turnip leaves over eight weeks of growth (normalized energy from LEDs).

The leaf counts for the experimental turnips are shown in Figure 8, and the normalized values are illustrated in Figure 9. All of the treatments exhibited a similar growth pattern, and the red light treatment produced more than double the leaf count when normalized. The small dip in counts in week 5 was due to some leaves dying out, which were quickly regrown.

**Figure 10:**
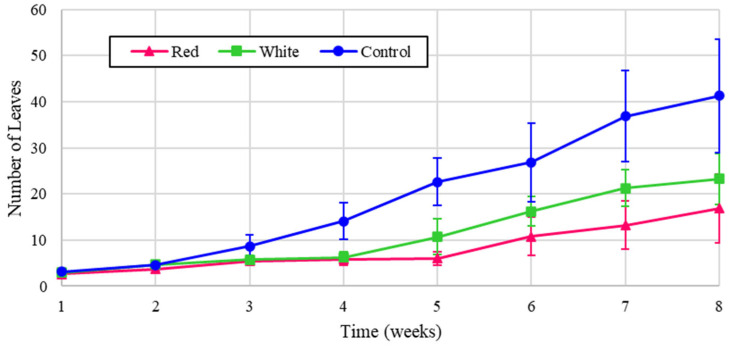
Number of radish leaves over eight weeks of growth.

**Figure 11:**
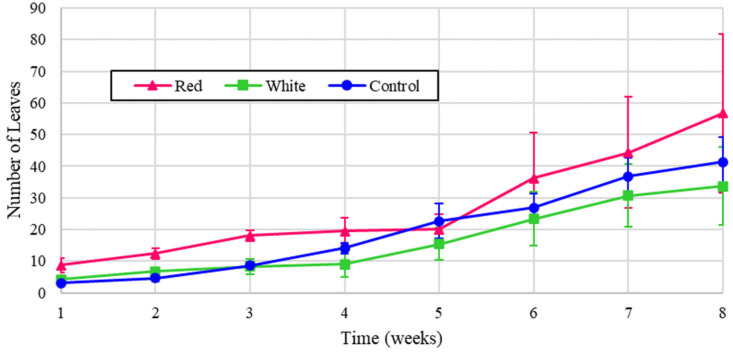
Number of radish leaves over eight weeks of growth (normalized energy from LEDs).

Similarly, the experimental leaf counts for the radishes are shown in Figure 10, and the normalized values are reflected in Figure 11.

Figure 10 shows that the radish leaves increased consistently with the amount of energy, demonstrating that light intensity has the most impact on growth, in agreement with [7]. The higher growth can be attributed to the control, white, and red light treatments, respectively. Similarly, as shown in the normalized values in Figure 11, the red light provided the highest leaf count. In Figure 11, however, all treatments possess very close values of leaf numbers, indicating the notable contribution of white light in increasing the fresh biomass of the studied cultivars.

### 3.3. Crop Yields

**Figure 12:**
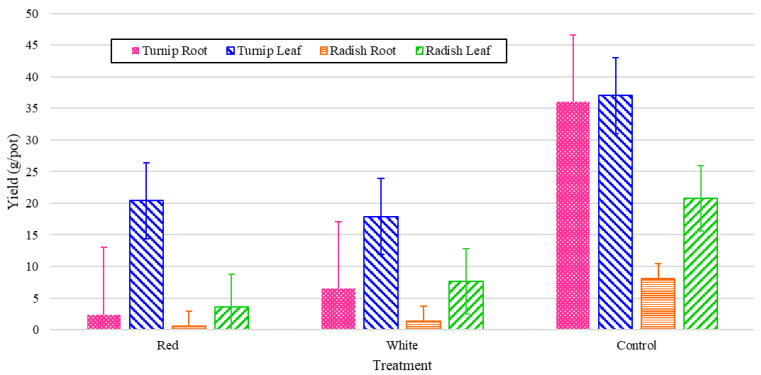
Yield values for turnip and radish fresh roots and green leaves grown under various light spectral treatments running for 24 h during the eight-week cultivation period.

The plants were harvested after eight weeks, having lived their entire lives from germination in the walls under 24 h light from their respective light treatments. The yields were recorded in grams per port (by averaging the harvested yield with respect to the total number of active ports) for both the edible leaves and for the root crops. The experimental values are shown in Figure 12, which shows that the turnips had much higher leaf and root yields compared to the radishes. In both cases, as can be seen in Figure 12, the leaf mass was greater than the root mass. As can also be seen, this trend was exaggerated in the white and red light treatments, which had substantially less energy shares. This can underscore the significance of other factors such as IR wavelength in the root mass growth. Figure 12 also shows that turnip leaf yields were high across all treatments, regardless of the wavelength of the total PPFD. Red light produced extremely low root growth for both the radishes and turnips, and while white outperformed both significantly for the turnips; only the control produced significantly higher radish root yields.

**Figure 13:**
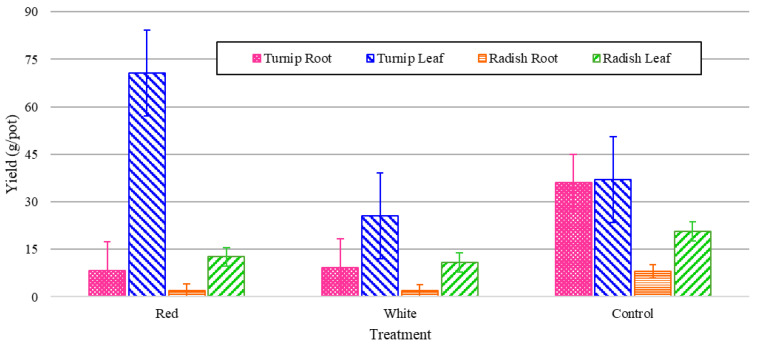
Yield values for turnip and radish fresh roots and green leaves grown under various light spectral treatments running for 24 h during the eight-week cultivation period (normalized energy from LEDs).

The normalized values in Figure 13 demonstrate that red light produces a much higher fresh biomass of turnip leaves than radish, with little significant effect on other relationships, likely due in part to the elongating properties of red light [11].

**Figure 14:**
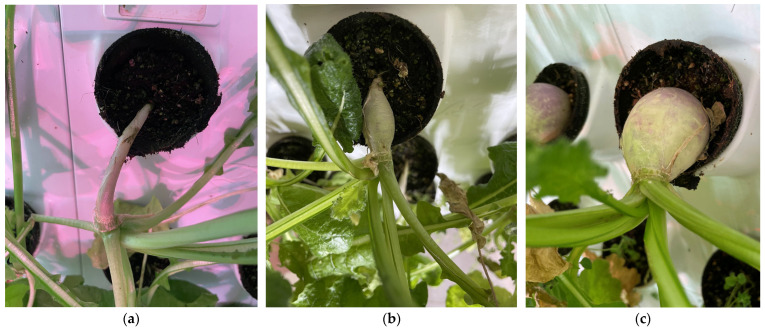
Representative turnip plants produced by each light treatment: (**a**) red light condition, (**b**) white light condition, and (**c**) control light condition.

Representative images of the turnip plants produced by each light treatment are shown in Figure 14. As can be seen in Figure 14, the growth of the roots is substantially larger under the full control light conditions, such that their biomass exceeded the capacity of the pots.

**Figure 15:**
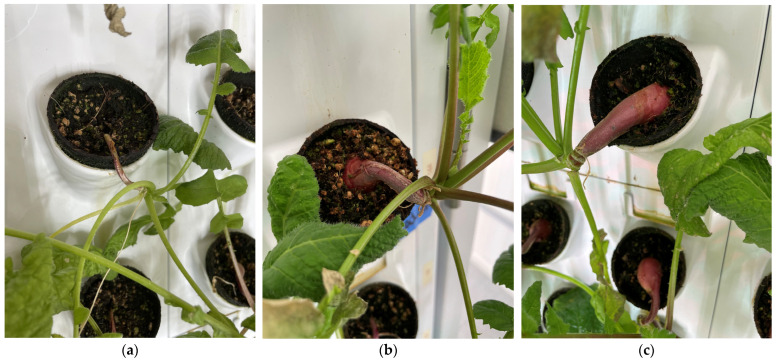
Representative radish plants produced under each light treatment: (**a**) red light condition, (**b**) white light condition, and (**c**) control light condition.

Similar results are evident in Figure 15 for the radishes.

**Figure 16:**
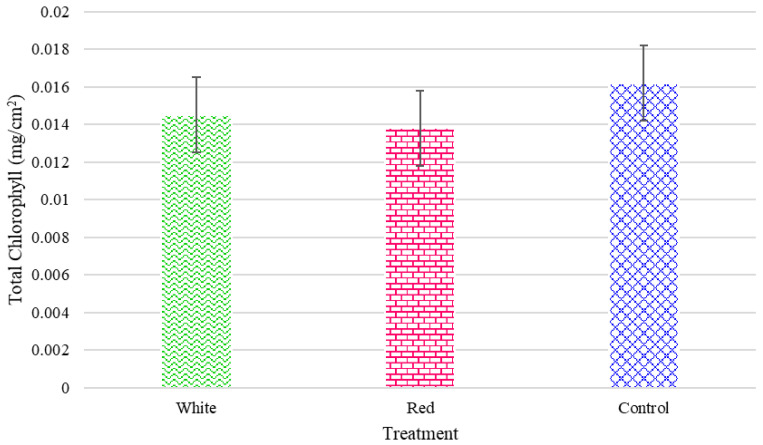
The total chlorophyll for the turnips grown under the three light treatments.

**Figure 17:**
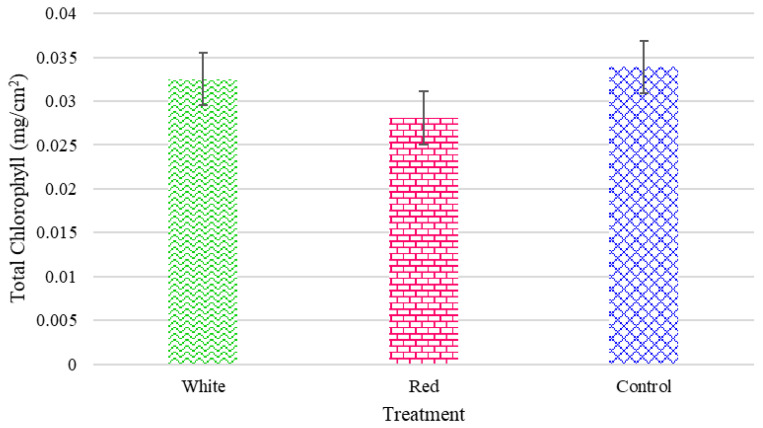
The total chlorophyll for radishes grown under the three light treatments.

The outcomes can in part be explained by the results of the measured chlorophyll content for the radishes (Figure 16) and turnips (Figure 17), respectively. The turnip and radish chlorophyll contents were not considerably affected by the light treatments, as illustrated in Figure 16 and Figure 17.

## 4. Discussion

Previous studies have heavily investigated the impact of light on crops. There have been many studies on leafy vegetables (romaine [21], spinach [17,22], chard [23], red salad [5,24,25], and kale [26]), which have all been covered in great detail. The results here are consistent, agreeing that leafy vegetables prefer a light quality ratio of 0.5–0.7 blue–red [27]. Basil and tomatoes have also been well researched in horizontal systems; basil thrives with 70% red light [28], and tomatoes similarly benefit from full-spectrum treatment [15]. Radishes tend to behave differently and do well with a higher ratio of red light [7], though purely red light is known to produce lower overall leaf mass due to the apparent role of petioles as sink organs, rather than roots [10,29]. Notably, the fresh root–shoot ratio obtained by Bukhov et al. was 0.17 under red light, which is similar to the result presented here under red light, which was 0.16, in close agreement. Thus, the results found here for a vertical system were consistent with the previous horizontal and vertical results on radishes and suggest that although normalized conditions show a high radish production under red light, their root growth is restricted. Overall, the radishes displayed the healthiest root–shoot ratio in the control conditions, and they clearly had a higher yield under the control conditions, confirming the high portion of red light required to grow radishes effectively. The yields were not particularly strong, as was expected for an indoor vertical farming facility, and this could be due primarily to the grow walls being designed specifically for growing greens.

This study, however, provides the first spectral light study for turnips in the literature and the first true indoor vertical growing study. The turnip results were much stronger than those of the radishes in terms of production of both roots and leaves. Turnip leaves can be sold in bunches for anywhere from USD 0.80 to USD 2.99 per bunch [30,31], depending on the location. They are often more expensive in northern states, as they are typically sourced from southern states [32]. According to normalized values, red light produces a large biomass of leaves; however, to maximize profitability, a higher root value should also be considered, as turnip roots can sell for around USD 0.43 per 100 g (current price at Walmart) [32]. For radish light treatment experiments, the effect of white light on the plant height, number of leaves, and chlorophyll (mostly green biomass indicators) was considerable according to the normalized energy data. It is worth noting that the control treatment did not vastly outperform the other treatments in either category, or it produced minor alterations when values were presented normalized against the consumed energy. The root yield of both turnip and radish, however, was significantly higher under full-spectrum conditions, even after normalization. In a low-energy environment, stress can shift growth [33], and plants may prioritize growing leaves to increase photosynthesis, causing roots to grow smaller (according to the yield values in Figure 12 and Figure 13).

Similar ongoing studies on the cultivation of organic greens in the agrivoltaics agrotunnel presents a reasonable ROI of over 10% for low labor rates [34]. Even organic or premium turnips and radishes are a much lower value crop; thus, the capital costs of the agrivoltaic agrotunnel would need to be under USD 200,000 to make a realistic profit, assuming no other major economic changes (e.g., food-related tariffs). Full economic evaluations should be carried out in future work, taking into account all cost expenditures (labor, consumable materials, and initial investments) associated with the cultivation of root vegetables. Further economic analysis is required to determine whether turnip leaf sales are profitable in an agrivoltaics-powered vertical layout or if they are better reserved for horizontal farming. If Northern U.S. or Canadian farmers were able to start suppling this using the agrivoltaics agrotunnel or similar growing systems, not only would transportation costs be reduced, but there would also be reductions in the environmental impacts. Full economic analysis on leafy greens has already been shown to be economic in such systems [34], and there is some evidence that because consumers support agrivoltaics [35], they may be willing to pay more for agrivoltaic crops [36]. This is particularly interesting because this form of agrivoltaics—where solar power is integrated to CEA—would allow for year-round production of these root vegetables and their green leaves. Since turnip leaves grow so abundantly under reduced lighting, these could be produced with much lower energy use than other products, which again would decrease capital cost because it would allow for downsizing of the PV array.

French breakfast radishes, which are typically much smaller, grew much closer to typical commercial size than turnips. This is primarily due to the limited root area available in cups optimized for leafy green production. The variety of turnips planted is best harvested at a diameter of two inches, and as the pots of the wall are also two inches, it is not possible to grow ideal turnip roots in this setup. Much larger grow bins exist for this lighting system [37]. It is possible, however, to have different sized ports on a grow wall, and container design is well known to influence growth [38]. To increase root growth, however, the radishes only thrived under full-spectrum lighting, so it would seem that the other treatments did not receive enough light to increase the photosynthetic rate of the plant to produce stronger roots. Future work can repeat these experiments using higher-intensity light of all three spectral ranges. It is worth noting that there was a significant difference for both turnip and radish root yield values between the white light and the control treatment. The addition of red and white light to the control, though it contributes little total PPFD, still has an impact on growth factors, allowing for a higher photosynthetic rate and greater production.

Though true energy normalization cannot be achieved due to the multitudinous effects of multiple wavelengths on plant growth, the normalized results are meant to predict what may occur should these plants be grown under pure lighting conditions with equal PPFD. It also shows how much contribution each wavelength had in boosting the growth indicators of the plants per unit of energy consumed. They show that the red light treatment could boost the growth of the plants’ green parts, e.g., leaf count and total weight. It is worth noting that very small amounts of white light were present in this treatment because the systems were not completely light impermeable (Figure 4), so the plants received small amounts of all wavelengths, which could also have impacted their scaled values. Additionally, controlling the photoperiod for the crops being grown would also save energy and can be targeted to maximize the growth. Although increasing the flux may be necessary for maintaining the optimal DLI, the required time period could be reduced. Reducing the light period down from 24 h/day has been shown to not impact the growth of some lettuces [34], but additional work is needed to determine if this is also the case for turnips and radishes. Finally, for turnips, there is already a need to improve the energy efficiency of its production to reduce carbon emissions [39]. Further analysis is required to determine whether turnip leaf sales are profitable in a vertical layout and if this approach using agrivoltaics coupled to CEA would reduce the overall energy and emissions for cultivation. A full environmental life cycle analysis could achieve this aim.

## 5. Conclusions

This is the first study to demonstrate that root vegetables including turnips and radishes could be successfully grown in an agrivoltaic agrotunnel using both lighting and grow walls optimized for cultivating leafy greens and salads [34]. As reductions in LED energy use are particularly important for agrivoltaic indoor vertical farming to minimize capital costs for PV modules, this study investigated the impacts of LED spectra on growth indicators of the studied cultivars. Both plants preferred more daily light integral or light intensity during the light operating hours than was available during 24 h with the LEDs designed for leafy greens. The normalized values, however, showed that they preferred to receive more red light to increase their height and number of green leaves. However, in some other cases, such as the number of radish leaves and total chlorophyll, the positive effect of white light was indisputable. For both cultivars, the leaves provided higher crop masses than the roots, although the turnips appeared to be far more adaptable to this approach than the radishes.

The results here show promise for providing true net-zero-energy root vegetables year-round, even in northern climates with agrivoltaics agrotunnels or similar PV-powered CEA. Future work is needed to optimize the grow walls with larger ports to allow for effective growth of these root vegetables and other crops. Further work with light intensity trials over the various wavelengths (e.g., including/excluding IR) is also required to reach optimal light recipes.

## Figures and Tables

**Table 1:** Total number of samples of each crop in each treatment.

Crop	Red	White	Control	Total
Turnip	10	11	10	31
Radish	14	12	11	37

## Data Availability

The original contributions presented in the study are included in the article, further inquiries can be directed to the corresponding author.

## Abbreviations

The following abbreviations are used in this manuscript:

CEA	Controlled Environment Agriculture
VF	Vertical Farming
LED	Light-Emitting Diode
EC	Electrical Conductivity
FSSC	Food Security Structures Canada
PPFD	Photosynthetic Photon Flux Density
PV	Photovoltaic
